# A Systematic Approach for Evaluating Artificial Intelligence Models in Industrial Settings

**DOI:** 10.3390/s21186195

**Published:** 2021-09-15

**Authors:** Paul-Lou Benedick, Jérémy Robert, Yves Le Traon

**Affiliations:** 1 Interdisciplinary Centre for Security, Reliability and Trust, University of Luxembourg, 6 Rue Richard Coudenhove-Kalergi, L-1359 Luxembourg, Luxembourg; Yves.LeTraon@uni.lu; 2 Cebi Luxembourg S.A, 30 rue J.F. Kennedy, L-7327 Steinsel, Luxembourg; jeremy.robert@cebi.com

**Keywords:** time series classification, artificial intelligence robustness, industrial internet of things, adversarial

## Abstract

Artificial Intelligence (AI) is one of the hottest topics in our society, especially when it comes to solving data-analysis problems. Industry are conducting their digital shifts, and AI is becoming a cornerstone technology for making decisions out of the huge amount of (sensors-based) data available in the production floor. However, such technology may be disappointing when deployed in real conditions. Despite good theoretical performances and high accuracy when trained and tested in isolation, a Machine-Learning (M-L) model may provide degraded performances in real conditions. One reason may be fragility in treating properly unexpected or perturbed data. The objective of the paper is therefore to study the robustness of seven M-L and Deep-Learning (D-L) algorithms, when classifying univariate time-series under perturbations. A systematic approach is proposed for artificially injecting perturbations in the data and for evaluating the robustness of the models. This approach focuses on two perturbations that are likely to happen during data collection. Our experimental study, conducted on twenty sensors’ datasets from the public University of California Riverside (UCR) repository, shows a great disparity of the models’ robustness under data quality degradation. Those results are used to analyse whether the impact of such robustness can be predictable—thanks to decision trees—which would prevent us from testing all perturbations scenarios. Our study shows that building such a predictor is not straightforward and suggests that such a systematic approach needs to be used for evaluating AI models’ robustness.

## 1. Introduction

Nowadays, with the advent of the Internet of Things (IoT) and Industrial IoT (IIoT), public and industrial actors are leveraging these technologies to enhance their systems while satisfying new requirements entailed by such a social revolution [1,2,3,4,5,6]. Focusing on the industrial context, with the industry 4.0 (r)evolution, companies want to meet new business goals by increasing the Overall Equipment Effectiveness (OEE). In the meantime, their customers are increasingly demanding better quality, flexibility or even security. In order to tackle these new challenges, industrial actors are looking at exploiting unused data coming from their production’s systems. The amount of data is unprecedentedly huge, making it difficult for humans to analyse and make decisions quickly and efficiently. That is the reason why Artificial Intelligence (AI) is being increasingly used for solving a large range of problems and applications, e.g., the Time Series Classification (TSC) problem, which is one of the most common in the industry and also recognised as one of the ten listed problems in data-mining researches [7].

Using AI requires good data quality whatever the applications. Although data-quality consideration strongly depends on the end-users or use-cases needs, it should be specifically considered for each data-driven system [8,9,10] to avoid regrettable experiences, e.g., a pedestrian killed by a self-driving car in Arizona [11]. Indeed, by learning from inaccurate or inadequate datasets, the downstream results can be flawed and lead to an inaccurate analysis of the data, resulting in inappropriate actions from the different actors [12,13,14]. In industry, there is a lot of situations where data quality can be degraded throughout the production system’s entire lifetime. Beyond the ageing of the sensors, the whole data collection infrastructure may introduce some perturbations. This is all the more true for companies with decades of existence that rely on legacy industrial architectures where data producers (i.e., sensors, Programmable Logic Controllers (PLCs), etc.) are heterogeneous, requiring middleware to access and standardise data, while providing an interface between the business and industrial worlds [15]. Such data collection infrastructure may have sparse performance, in particular in terms of network metrics such as losses, delays or traffic-load [16,17], resulting in data quality degradation.

Unfortunately, most of the studies—tackling the TSC problem using AI—do not consider such data quality degradation scenarios when evaluating their algorithms. Researchers are indeed focusing on improving the algorithms performance especially in terms of accuracy or even response time [18]. Performances are evaluated on public benchmark datasets such as the UCR datasets ([19] (https://www.cs.ucr.edu/%7Eeamonn/time_series_data_2018/, accessed on July 2021)). Such datasets are then considered as clean and without any biases. Although these evaluations are needed, this is not sufficient to be confident in the robustness of these algorithms/models in case of perturbations (that may happen temporarily or gradually over time).

The objective of the paper is therefore threefold: (i) to propose a systematic approach, inspired by mutation testing techniques, for artificially injecting two types of perturbations in benchmarking datasets, (ii) to evaluate the impact of such perturbations on 7 state-of-the-art algorithms and 20 sensor-based datasets and (iii) to analyse whether such impact can be predictable or not without testing all perturbations scenarios.

The paper is organised as follows: Section 2 presents the related work regarding AI that consider perturbations. In Section 3, a systematic approach for evaluating AI models under perturbations is developed. Then, in Section 4, the systematic approach is therefore assessed by experiments on two realistic perturbations, called hereafter swapping and dropping perturbations. Section 5 aims at trying to predict the robustness of models by using decision trees. Finally, Section 6 presents the conclusion of the paper.

## 2. Background and Related Work

AI is a domain that includes a lot of techniques to tackle a large range of problems and applications. Focusing on the *Time Series Classification problem*, Machine-Learning (M-L) techniques are beginning to be the new standard in recent industrial systems, and Deep-Learning (D-L) also tends to be adopted in certain cases ([12,20,21,22]). Looking at the definition of a time-series (TS) in the literature, authors use different ones depending on the context [18,23,24] while being quite similar. It is worth mentioning that it is, nonetheless, important to define it, as pointed out in [25]. In this paper, we define it as follows:


A time series TS is an ensemble *E* representing a sequence of *N* data-points $e_{n}$, assumed as
equally distributed: $E=[e_{1},\cdots ,e_{N}]$.

Our literature review is intended to analyse to what extent research work (in M-L and D-L) are evaluating “the degree to which a system or component can function correctly in the presence of invalid inputs or stressful environmental conditions”, defined as robustness in IEEE standard glossary of software engineering terminology [26]. In the AI context, “robustness (therefore) measures the resilience of an M-L system’s correctness in the presence of perturbations” [27]. Based on these definitions and the aforementioned literature review objective, we applied the following three-step methodology for selecting papers to analyse: (i) keep only papers dealing with the TSC problem. Our corpus consists of 1417 papers collected in seven main library databases: IEEE Xplore, ACM Digital Library, Springer, ScienceDirect, MDPI, Taylor and Francis and Wiley; (ii) filter papers mentioning perturbations (or related terms, e.g., robustness, adversarial, data inconsistency). Only 35 papers were remaining, and (iii) they were filtered through a careful reading. Finally, 14 papers are presented and listed in Table 1, while summing up (as in Table 1 footer) with:

- column “Approach”: the approach (M-L or D-L) used in the research work;
- column “Method”: the methods/algorithms used or analysed;
- column “TS Type”: the type of time-series (TS), i.e., either Univariate Time Series (UTS) or Multivariate Time Series (MTS);
- column “Perturb. Model”: the type of perturbations model;
- column “Reproducible?”: if such analysis is reproducible (can we recreate ourselves datasets with perturbations according to predefined parameters);
- column “Public repo?”: if such datasets before/after perturbations are publicly available.

This highlights that there are only a few works that evaluate the robustness of models by providing a reproducible model of their (natural) perturbations or a (artificially) perturbed dataset that is publicly available. Only 3 studies (i.e., [29,32,40]) out of 14 (artificially) generated perturbations to modify the datasets and then perform experiments on the models. Other listed studies do not provide a fault model that is reproducible, or they use datasets that are known as containing some perturbations (noise or missing data) but without identifying the characteristics of these perturbations (making it difficult to reproduce on other datasets for comparison purposes). Concerning the studies in which perturbations’ models aim at modifying the data, the experiments are focused on the perturbations models and evaluating few algorithms, but they do not analyse to what extent the perturbations impact the performance of the model itself. Moreover, even if research solving some adversarial robustness problems exists, adversarial robustness can lead to a decrease in accuracy when no perturbations are present ([42]).

Based on those facts, we decided to study the robustness of M-L/D-L models under perturbations, which we defined in Section 3 and allows anybody to reproduce them for benchmarking purposes. To do so, we selected two different works as the baseline:

- The algorithm presented in [18], called The Word ExtrAction for time SEries cLassification (WEASEL)—as an M-L solution, which obtains the best accuracy of the former algorithm on most of the public UCR datasets,
- The framework/algorithms presented in [24], which can be used as a black-box in the D-L category and showed great performance on UCR datasets.

In addition, as we focused on industrial scenarios, only sensor-based datasets (UTS) were used for our experiments (20 in total, presented in the following section).

## 3. A Systematic Approach for Evaluating AI Models under Perturbations

In this section, we intended to define a systematic approach for evaluating AI models under perturbations that could appear over the AI models’ lifetime. In that sense, we assume that the AI models are trained in “normal conditions”—it does not mean that the data are clean and without any biases but reflects the normal behaviour of the data collection infrastructure at a time of the model training. Usually, test datasets are also collected in the same conditions—even if it is not clearly mentioned, the characteristics of the training and testing datasets are similar. There is an important literature in ML that demonstrates that trained models may not be robust to corner cases (e.g., adversarial cases) despite a high accuracy. We thus believe that trained models shall be tested against perturbation to assess and improve their robustness in situations that may occur in a realistic setting. It also important to include potential derivations in the data that could appear over time or in “degraded conditions”, so as to evaluate their robustness. Our objective is therefore to generate perturbations (on test datasets) that are not too far from the reality and more importantly reproducible—either on the same datasets or in a similar way on other datasets—so as to be able to benchmark/compare the robustness of AI models under such perturbations. In this approach, we define two kinds of perturbations:

- *The swapping perturbation*: the sequence of the *N* data points $e_{n}$ is altered/not respected. It is a realistic situation in several settings, e.g., when using User Datagram Protocol (UDP) as transport protocol for data exchanging between sensors and controller, since UDP does not enable re-ordering of the packets in the network and/or if the timestamping of the data can only be performed on the controller side (sensors usually do not have the capacity of timestamping).
- *Dropping perturbation*: some data points in the time-series are missing. As for the swapping perturbations, this is realistic as a network protocol such as UDP does not enable packet retransmission in the case of loss, or when software processing data reception has memory overflow (especially when processing a huge amount of sensors data in constrained devices, such as raspberry-like devices), which can also lead to such losses.

Since every (industrial) environment is different, it is usually difficult to identify the perturbations existing in such environment. That is the reason why our perturbations require to be parameterised using a suitable mathematical and systematic form. It enables, in particular, varying the parameters so as to identify the limits of the robustness of the AI models in the experimental analysis. As a matter of fact, let us formally define the perturbations:

- *The swapping perturbation*: Let us first reiterate that a time series TS is an ensemble *E* representing a sequence of *N* data-points $e_{n}$, assumed as equally distributed: $E=[e_{1},\cdots ,e_{i},\cdots ,e_{j},\cdots ,e_{N}]$. A swapping perturbation is therefore a pair of data points that are interchanged/swapped. In the case where only one pair has been swapped, the time-series becomes $E^{\prime }=\left[e_{1},\cdots ,e_{j},\cdots ,e_{i},\cdots ,e_{N}\right]$, where the events $e_{i}$ and $e_{j}$ have been swapped. However, swapping only one pair would probably not have an impact. We therefore define two parameters:
  – Pe as the percentage (0%<Pe<100%) of swapped events/values in each time-series of a dataset. It means that $S=N*\frac{Pe}{100}$ values will be randomly interchanged in a TS of length *N*.
  – *R* as the range in which the value is swapped (i.e., at which position the data are moved in a certain range of possibilities). For instance, let R=[1,2], which means that if we randomly pick 1. as the position to be changed, an event $e_{4}$ is interchanged with the event $e_{3}$ (and vice-versa).

To apply such swapping perturbations on all the time-series in a dataset *D*, we define the function presented in Algorithm 1, which gives a new dataset $D^{\prime }$ as the output. Note that a dataset *D* consists of a set of times-series $E_{k}$ with the same length—i.e., the number of data points *N* (as it is usually the case in public benchmarking repositories)—, such as $D=[E_{1},\cdots ,E_{M}]$ with *M* the number of TS in *D*. The newly created dataset $D^{\prime }$ consisting of a set of times-series $E_{k}^{\prime }$ has the same features (in particular, the number of TS and of data points per TS) than *D*. Finally, to keep it as generic and open (for experimentation) as possible, no probability distribution is imposed in the random processes used in this algorithm.

**Algorithm 1.** Swapping perturbations function.   **input**: *D*, Pe, *R*
   **output**: $D^{\prime }$

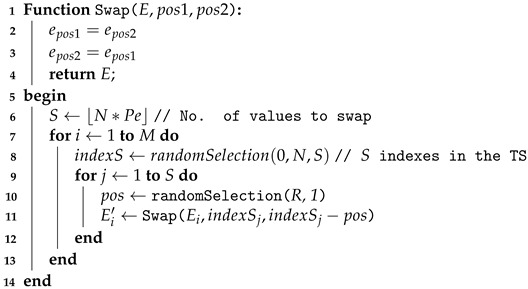

- *Dropping perturbation*: A dropping perturbation is the consequence of a data loss (e.g., between a sensor that has sent the data to be stored and the controller that has to store the data). Formally speaking, it means that a time-series $E=[e_{1},\cdots ,e_{N}]$ is becoming $E^{\prime }=\left[e_{1},\cdots ,e_{N-Q}\right]$ where the length of the time-series is decreased in terms of the number of lost/deleted events *Q* (Q<N). However, in that case and particularly in practice, $E^{\prime }$ will not strictly follow the definition of a TS where the data points $e_{n}$ are assumed as equally distributed over time. This means that all the indexes will be only shifted. To be consistent, mathematically speaking, we propose to have a reconstruction mechanism to fill out a missing value (e.g., in practice, the controller knows that it should receive a value periodically, so it can compute a value when detecting a missing value). Even if such mitigation mechanism can limit the impact on the models’ robustness a priori, it is important for us to consider it in a formal way, since in a real environment, it would be probably and easily implemented. We nonetheless keep the term “dropping” since it is the origin of the expected perturbations. Based on these choices, we define a dropping function presented in Algorithm 2. It also takes the percentage Pe of removed events/values in each time-series of a dataset *D* as a parameter. Similarly to the swapping function, the indexes of the dropped (and reconstructed) elements are randomly selected. Note that, after reconstruction, the length of $E^{\prime }$ is equal to the one of *E*. Finally, to keep it as generic and open (for experimentation) as possible, the method for recomputing a value at the dropped values positions is not imposed by the algorithm as such.

**Algorithm 2.** Dropping perturbations function.  **input**: *D*, Pe

  **output**: $D^{\prime }$

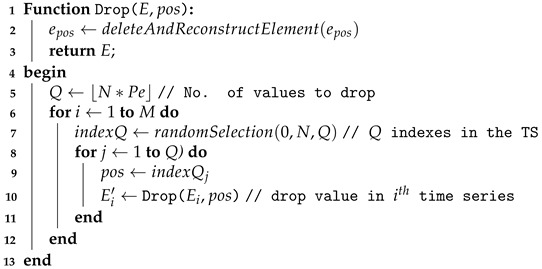



## 4. Robustness Evaluation Based on Perturbations Generation

The approach presented in the previous section is applied for evaluating the robustness of AI models trained with seven algorithms: six D-L algorithms (referred to as Fully Convolutional Neural Network (FCN), Residual Network (ResNet), Multi Layer Perceptron (MLP), Multi-Scale Convolutional Neural Network (MCNN), Multi Channel Deep Convolutional Neural Network (MCDCNN) and Time Le-Net (Tlenet)) proposed as a framework by Fawaz et al. [24] and one M-L algorithm (called WEASEL) developed and evaluated by Schäfer and Leser [18]. This evaluation is achieved on 20 sensor-based datasets available in the public UCR (https://www.cs.ucr.edu/%7Eeamonn/time_series_data_2018/, accessed on 5 July 2021) repository. These were selected since the data looks similar to the ones we can find in industrial scenarios, when a sensor delivers a univariate time series. The algorithms are selected for two reasons: (i) they are freely and publicly available to be used as a blackbox, and (ii) they give good performance on the selected datasets to be served as a baseline of our work.

### 4.1. Methodology of the Evaluation

Let us first present the methodology used for this evaluation:

1. The preparation phase consists of training models with the available datasets and then generating datasets with perturbations that will be used for evaluating the robustness of the models in phase 2.
  (a) Retrieve the 20 selected univariate datasets of the sensors’ type (from the UCR repository).
  (b) Train models using the different selected classifiers on the previously collected datasets. This phase is needed since the models (of existing researches) for the benchmark datasets are not available publicly (and the hardware as well as the software can impact the models’ accuracy, especially when D-L is used). Since training a D-L model several times can lead to different accuracy results (even with the same parameters and dataset), we trained each pair 5 times <classifier; dataset> (it was enough to reach the same—or even better—accuracy as the existing benchmark). In total, 700 models (5 iterations for 7 classifiers on 20 datasets) were trained. In this training phase, the objective is to obtain the best classifier/model that could be deployed in real settings. In that sense, we kept only the best iteration/model for each pair <classifier; dataset>, resulting in 140 models.
  (c) As a first filter of our evaluation, we keep only the models that have an accuracy higher than 90% (models with lower accuracy would not even be considered for deployment in practice, or even for trying to improve them before deployment) on test datasets without perturbations. Results of this step are given in Table 2, where results in red are related to the models we used in the following (53 models). In this table, two accuracy values are presented for each dataset. This represents (i) the accuracy results obtained in the literature (column ‘Ref’)—especially in [24]—and (ii) our own accuracy results (column ‘Our’). As explained previously, the hardware (as well as the software) can impact such results, so comparing results enables only keeping models that have equal or greater accuracy than the ones in the literature. Note that, as the Tlenet classifier does not offer satisfying models for any datasets (i.e., with accuracy >90%), it will not be studied any deeper. Similarly, no classifier gave suitable results on the datasets ‘DodgerLoopDay’, ‘DodgerLoopGame’, ‘Earthquakes’, ‘FordB’, ‘InsectWingbeatSound’, ‘Lightning2’ and ‘Lightning7’. These datasets will, therefore, be discarded from further analysis.
  (d) Generate new datasets containing perturbations as defined in the previous section. In this evaluation, we generated a total of 13,250 DS (5 different values for Pe—from 1% to 20%, in steps of 5%—using swapping and dropping perturbations and 9 different values for *R* using swapping perturbations from 1 to 10 positions in steps of 1, all over 5 iterations to take into account the randomness of the perturbations functions). Note that only uniform distribution has been used in the random processes, and a linear regression between the previous and next values (i.e., the average value: $e_{j}=\frac{e_{j+1}-e_{j-1}}{2}$, where *j* is the index of the dropped/reconstructed value) is used for filling out the dropped value. Note also that this linear regression used to reconstruct a dropped value is convenient for the implementation of the D-L algorithms since it requires the same length for all the TS (in addition to following the mathematical properties of a TS, i.e., to be equally distributed). We therefore believe that practitioners need to be aware of such constraints when implementing AI models.
2. The empirical study consists of:
  (a) Evaluating the robustness of each model (on each dataset generated);
  (b) Concluding about the impact of such perturbations on the algorithms/models.

To run these experiments, we implemented the D-L models by using Keras 2 (https://keras.io, accessed on July 2021) framework with TensorFlow backend (Python 3.6) and the WEASEL algorithm developed in Java. All the models were trained on the University of Luxembourg High-Performance Computer (HPC) with 1 Graphics Processing Unit (GPU) (NVIDIA TESLA V100) on Compute Unified Device Architecture (CUDA).

### 4.2. Results of the Evaluation

**Figure 1:**
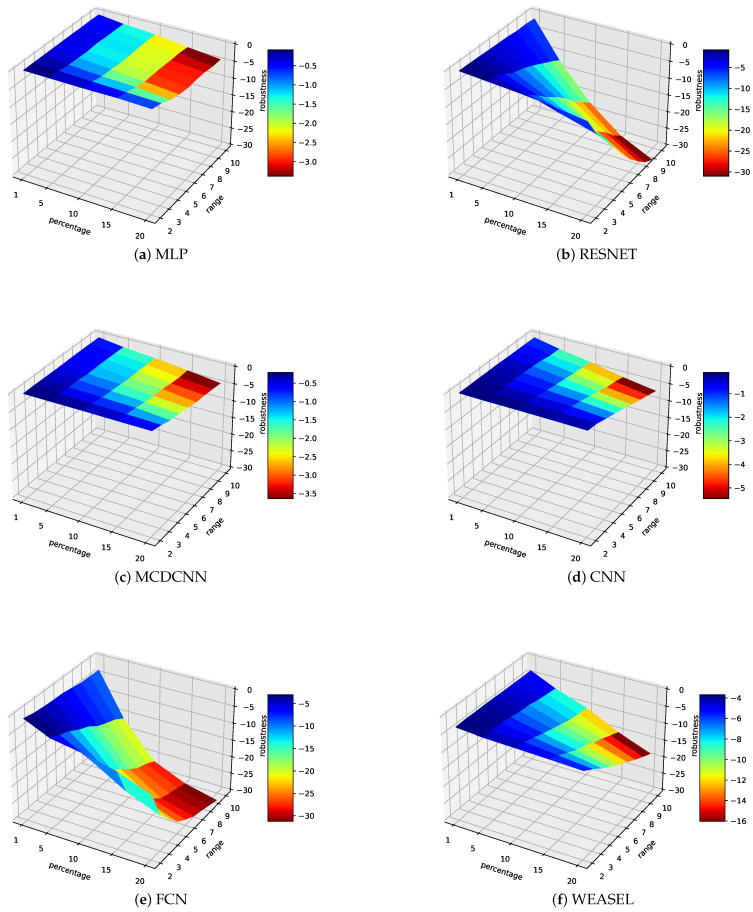
Average robustness over all the considered datasets under swapping perturbations.

Let us now look at the results of the empirical study with regard to the perturbations. Table 3 and Table 4 present an overview of our results. For the sake of our analysis, we consider here that a model is impacted if its accuracy decreases more than 1% over the experiments (with regards to its accuracy without any perturbations). Results show that:

- Very few models are not impacted at all by the swapping perturbations. To understand to what extent the models are impacted by such perturbations over the considered sensor-based datasets, we compute an average robustness, as shown in Figure 1. It appears that:
  – *MLP, MCDCNN, CNN*: Even if the results of the MLP, MCDCNN and CNN models are limited to, respectively 4, 5 and 7 (out of 13 possible models/ datasets), the impact of these perturbations on the models tends to be quite limited since in the worst case (i.e., with a percentage of 20% and a swap range of [1–10]), the loss of accuracy is, respectively, around 3%, 3.5% and 5% on average. Of course, in practice, the tolerance of such degradation would require analysis for each given use case.
  – *RESNET, FCN*: Contrary to MLP, MCDCNN and CNN, RESNET and FCN have been evaluated on all possible models of our study. This analysis shows clearly that such algorithms/models are more rapidly impacted by the swapping perturbations since the loss of accuracy is already about 10% on average for a low percentage of swap values (5%) and a small range ([1–3/4]) to reach 30% in the worst case scenario of our study.
  – *WEASEL*: Although WEASEL is clearly impacted by the swapping perturbations, the impact is more limited (compared to RESNET and FCN) for low percentage and range since the loss of accuracy tends to be less than 6% before being really degrading when the percentage is important (more than 15%) and/or range is high (more than [1–4]).
- Very few models are impacted by the dropping perturbations. This shows how the mitigation mechanism (as simple as it is) plays an important role in the models’ robustness. It is therefore really important that practitioners understand this point and integrate it from the design phase of such AI usage. Note that, even if we consider RESNET impacted on the Car dataset, the accuracy decreases only by 5% in the worst iteration (over the 5) when the perturbations are at the maximum (i.e., $P_{e}=20\%$). Similar behaviour was found for FCN on this dataset (but from $P_{e}\geq 15\%$). FCN accuracy on FordA decreases by 6% and less than 2% on SonyAIBORobotSurface1 in the worst iteration. Weasel on FordA appears to be an exception where the accuracy is decreased by 21% to 41%.

Overall, this study demonstrates that some of the models/algorithms can be impacted by the data quality, especially when it is decreasing over time. Indeed, the conditions in which the data are collected can be different from the time the models were trained, leading to an accuracy decrease. Based on those results, one may wonder whether such degradation can be predicted before deploying a model in real-life scenarios, i.e., without the need to generate as many datasets as possible for testing it under perturbations (as proposed by our systematic approach). The next section tries to answer this question.

## 5. Is Robustness Predictable?

The objective is to answer the question, is robustness predictable? If so, we aim to provide humans/engineers with a method—as simple to understand and to interpret as decision trees—to determine whether a model will be impacted by some of the perturbations. To do so, we assume that the characteristics of the datasets (i.e., the shape) impact the robustness of the models. Based on this assumption and our previous results, we created a dataset (consisting of 2700 rows) with the following information:

- The characteristics of the time-series of each dataset presented in Table 5, i.e.,
  – The time-series length (denoted len(TS),
  – The number of classes ($No_{cl}$),
  – The Pearson correlation coefficient [43], which is defined as the covariance of two variables divided by the product of their standard deviations. It is an intuitive and easy to understand a way of measuring the linear correlation between two signals (here, two time-series), which has been used in many studies from different fields to characterise the correlation between time series (Adler and Parmryd [44], Benesty et al. [45]). In this study, we used the average (i) of the Pearson correlation coefficients computed between all time series of the same class ($P_{in-cl}$) and (ii) of the Pearson correlation coefficients computed between the different classes for all the datasets ($P_{bet-cl}$).
  – The average derivative (Deriv), enabling to reflect the changes/variations in a time series.
- The parameters of the considered perturbations as defined in Section 4, i.e., $P_{e}$, the percentage of swapped/dropped values, and *R*, the range in which the value is swapped,
- Finally, a label (per classifier) representing if the model is impacted (or not) by such perturbations/characteristics settings.

This dataset is used to create as many decision trees as classifiers. To do so, we used the sklearn decision trees library (https://scikit-learn.org/stable/modules/tree.html, accessed on June 2021). Although one of the major features of the decision trees visualisation, the size and number of our decision trees are too significant to be presented here. Note that the StarLightCurve dataset does not appear due to the computational resources that are needed to compute the different parameters since it contains too many long time series. Table 6 gives an overview of the number of leaves and depth. This shows an important disparity between the decision tree’s features, and more particularly, the number of leaves, e.g., MLP has “only” 21 leaves while resnet has 125 for the swapping perturbations. The depth is relatively steady, even if it is quite important with 7 to 12 levels. Contrary to the decision trees for the swapping perturbations, their number of leaves and the depth for dropping perturbations are not so important (even very low). Indeed, several classifiers count only 1 leaf and a depth of 0, showing the ability of models to correctly classify the time series after perturbations as it has also been raised in the previous section (partially due to the linear regression mitigation mechanism). Overall, such decision trees (in particular for swapping perturbations) do not generate easy-to-understand rules as we expected and do not provide clear indications of which parameter(s) impact the robustness the most (or the classification in a class ‘impacted’/‘not impacted’).

**Figure 2:**
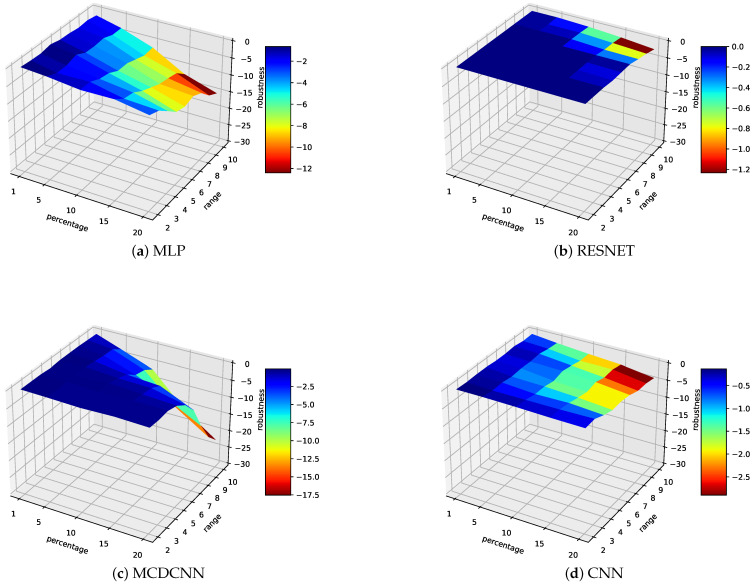

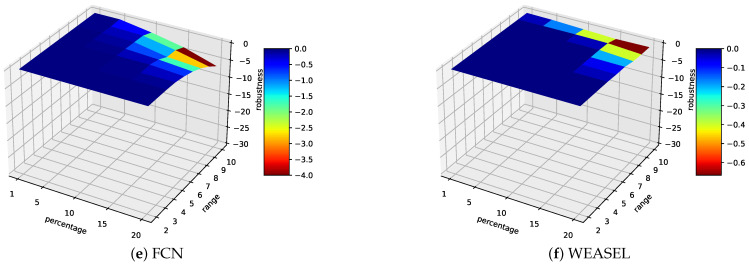
Average robustness over FischerTecknik dataset under swapping perturbations.

To test if such decision trees are nonetheless applicable to predict whether the model accuracy will be impacted by our perturbations, we applied a proof by contradiction (reductio ad absurdum), assuming that the characteristics of the datasets and therefore the decision trees enable predicting the impact of the perturbations on a dataset. To put it in another way, if an example does not satisfy this assumption, then the answer to the aforementioned question will be considered as ‘No’. To do so, we developed a case-study for collecting our own data. Thanks to our Fischertechnik factory simulation (https://www.fischertechnik.de/en/service/elearning/simulating/fabrik-simulation-24v, accessed on July 2021), we collected data from a light sensor that is used for classifying the parts according to their colours (blue, white or red), i.e., 3 classes for our time-series classification problem. The dataset’s characteristics are described in the Table 5. Note that the training and testing sets consist of, respectively, 100 and 50 TS of each colour (i.e., resp., a total of 300 and 150 TS). New datasets with perturbations have, therefore, been generated as achieved with the public datasets (cf. previous section) and analysed similarly, as shown in Figure 2. Then, decision trees are applied to the characteristics of our original datasets to predict if the model will be impacted with a given level of perturbations. Table 7 gives an overview of the results. Overall, this shows that:

- *Swapping perturbation*: we notice a disparity in the results between D-L and M-L methods.
  – *Deep-learning*: The accuracy of the decision trees on deep-learning models is really low. This means that the decision trees here are not able to predict whether the dataset will be impacted (or not). Indeed, by training decision trees, we try to create a model (the tree) that represents the behaviour of the D-L classifier. However, deep-learning models are very complex. There are so many parameters to take into account—even the hardware resources—, which make it almost impossible to predict its behaviour beforehand. To illustrate this complexity, let us look at Figure 1 where Resnet, FCN and Weasel were the least robust classifiers under perturbations over the 12 datasets, while MLP, MCDCNN and CNN seemed to be robust. This observation could have led to a first—quick/natural-–conclusion, where the three latter classifiers should be the best to deploy (especially in a possibly noisy environment). However, regarding the results of our case-study, the results are the opposite: MLP, MCDCNN and CNN are the classifiers for which the accuracy decreases quicker under perturbations when Resnet, FCN and Weasel are more robust to them. One may note that this has further opened up a research topic on explainable M-L.
  – *Weasel*: Concerning Weasel, which is more a ‘traditional Machine-Learning classifier’, a decision tree is more able to predict the impact of a future perturbation. Actually, the decision tree has an accuracy of 89%, which can be satisfying for helping humans to make a decision out of it (especially when associated to his/her expertise of the environment).
- *Dropping perturbation*: decision trees have a better accuracy in such perturbations. This is due to the small impact they have on the robustness of the models (again, thanks to linear regression mitigation mechanism), resulting in few scenarios where models are impacted, leading to an easier behaviour prediction.

In conclusion, this study shows that it is not easy to predict (based on the characteristics of a dataset) that some perturbations will impact the accuracy of a model trained on a dataset assumed to be ideal (i.e., without perturbations). As a consequence, the systematic approach, presented in Section 3, is really important to perform for evaluating AI models under perturbations before the deployment in an industrial environment prone to data quality degradation.

## 6. Conclusions, Implications, Limitations and Future Research

### 6.1. Conclusions

The world is engaging its digital transformation by providing industry with new tools for controlling their production and business systems. It aims at improving the efficiency of the production while covering the needs of sustainability, transparency, traceability and customisation requested by the customers. Thanks to the huge amount of sensor data available, AI is suitable for decision-making. However, in many companies, there is a lack of skilled engineers who master both AI technologies and the business specificities of the company. Thus, a condition for broad adoption is that engineering a performant and robust AI-based system must remain simple while leading to performances at least equal to existing solutions. In that sense, industries are not ready to implement such technology without being convinced that it will work smoothly and properly. That is why it is important to also evaluate the performance of AI models under perturbations (that could happen in the industrial environment). This paper shows that it is costly and hardly predictable, since predicting whether an AI model will be impacted is not straightforward (or not accurate enough). This shows the necessity to generate different perturbations (as presented in this paper) to evaluate the robustness of the AI models.

### 6.2. Implications

This research presents two main implications. First, it points out that M-L/D-L researchers should not stop their model evaluation once they have the accuracy computed on clean datasets and without any biases. The robustness of their model should always be assessed by presenting the perturbations formally. Second, it shows practitioners that simple measures such as the linear regression used for handling potential losses (dropping perturbations) can prevent AI algorithms from degrading in noisy environments.

### 6.3. Limitations and Future Research

Some limitations of our research can be pointed out. First, the approach and the empirical study are only based on two kinds of perturbations (swapping and dropping perturbations). Future researches will go further in that direction by proposing analyses of other perturbations (which we encourage when implementing/evaluating such an AI system in/for such industrial environment). In addition, choices have been made concerning the range of perturbations’ parameters. For instance, ranges and percentages of perturbations could be widened, or a finer-grained analysis could be conducted. Furthermore, our perturbations follow a uniform distribution, which can be adapted for other use-cases. Finally, the proposed methodology for predicting the robustness of the AI models, relying on decision trees, is based on “only” the experiments of thirteen datasets and seven algorithms for each kind of perturbation to train the trees. This might be too few, and input training data could give different results with more inputs.

## Figures and Tables

**Table 1:** Related work.

Paper	Approach		Method	TS Type		Perturb. Model	Reproducible?	Public Repo?	
	**M-L**	**D-L**		**UTS**	**MTS**			**Initial**	**Modified**
[28]	✗	✓	CNN	✓	✗	Random Noise	✗	✗	✗
[29]	✓	✗	SVM,1-NN, DT, RF	✗	✓	Data Loss	✓	✓	✗
[30]	✓	✗	XG-Boost	✓	✗	Missing Data	✗	✗	✗
[31]	✓	✓	SVM, DT, RF, NN, CNN	✓	✗	Random Missing Data	✗	✗	✗
[32]	✗	✓	ResNet, FCN	✓	✗	Noise	✓	✓	✗
[33]	✗	✓	CNN	✓	✓	Missing Data	◗	✓	✗
[34]	✓	✗	ARM-SONS	✓	✗	Missing Data	✗	✓	✗
[35]	✓	✗	BPSO, IBPSO, INSIGHT	✓	✗	Random Noise	✗	✓	✗
[36]	✓	✗	DTW	✓	✗	Missing data and Noise	◗	✓	✗
[37]	✓	✗	ANN, SVM, SSL	✗	✓	Noise	◗	✓	✗
[38]	✓	✓	OSVM, DNN	✗	✓	Colour Perturbations	✗	✓	✗
[39]	✓	✓	STRiD, NN, SVM, ID3	✗	✓	Missing Data	◗	✓	✗
[40]	✓	✗	1-NN	✓	✓	Missing Data	✓	✓	✗
[41]	✓	✗	BoW+SVM	✓	✗	Noise and Artifacts	◗	✓	✗

✓: yes, ✗: no, ◗: original dataset contains the perturbation, “Approach”: the approach (M-L or D-L) used in the research work, “Method”: the methods/algorithms used or analysed, “TS Type”: the type of time-series (TS), i.e., either Univariate Time Series (UTS) or Multivariate Time Series (MTS), “Perturb. Model”: the type of perturbations model, “Reproducible?”: if such analysis is reproducible (can we recreate ourselves datasets with perturbations according to predefined parameters), “Public repo?”: if such datasets before/after perturbations are publicly available, CNN: Convolutional Neural Network, SVM: Support Vector Machine, 1-NN: 1- Nearest Neighbors, DT: Decision-Tree, RF: Random Forest, XG-Boost: eXtreme Gradient Boosting, NN: Neural Network, ARM-SONS: Sparse Online Newton Step for AR with Missing Data, BPSO: Binary Particle Swarm Optimization, IBPSO: Immune Binary Particle Swarm Optimization, DTW: Dynamic Time Warping, ANN: Artificial Neural Network, OSVM: One-class Support Vector Machine, SSL: Semi-Supervised Learning, DNN: Deep Neural Network, STRiD: Statistical Tolerance Rough Set induced Decision tree, ID3: Iterative Dichotomiser 3, BoW: Bag of Words.

**Table 2:** Accuracy of the best models for each dataset (accuracy in %).

Dataset	mlp		Resnet		Tlenet		Mcdcnn		cnn		fcn		WEASEL
	Our	Ref	Our	Ref	Our	Ref	Our	Ref	Our	Ref	Our	Ref	
Car	77	80	**93**	**93**	32	32	75	80	78	80	**93**	**93**	82
DodgerLoopDay	54	16	54	15	16	16	54	53	59	58	40	15	53
DodgerLoopGame	86	88	86	80	52	48	88	90	83	83	78	78	80
DodgerLoopWeekend	**99**	**98**	**96**	**96**	74	74	**99**	**99**	**98**	**98**	**91**	**93**	**97**
Earthquakes	76	73	75	73	75	75	75	75	72	72	74	73	74
FordA	85	82	**94**	**95**	52	52	89	89	90	90	**92**	**92**	**97**
FordB	72	71	82	82	50	50	70	73	77	77	78	78	83
FreezerRegularTrain	82	**91**	**100**	**100**	50	50	**98**	**98**	**99**	**99**	**100**	**100**	**98**
FreezerSmallTrain	69	69	**96**	**93**	50	50	70	74	74	75	71	71	**91**
InsectWingbeatSound	66	61	51	50	9	9	61	58	59	59	40	40	63
ItalyPowerDemand	**96**	**96**	**96**	**96**	50	50	**97**	**97**	**96**	**96**	**96**	**96**	**96**
Lightning2	77	70	80	80	54	54	72	69	67	66	77	75	61
Lightning7	67	64	85	85	26	26	62	64	70	66	82	84	70
MoteStrain	87	86	**94**	**93**	54	54	85	86	89	**90**	**94**	**94**	**95**
Plane	**96**	**98**	**100**	**100**	14	14	**98**	**98**	**98**	**97**	**100**	**100**	**100**
SonyAIBORobotSurface1	73	70	**97**	**97**	43	43	79	**90**	71	72	**97**	**97**	85
SonyAIBORobotSurface2	83	83	**98**	**98**	62	62	84	86	84	84	**98**	**99**	**95**
StarLightCurves	85	**95**	**98**	**98**	58	58	**95**	**95**	**93**	**93**	**97**	**97**	**98**
Trace	61	81	**100**	**100**	24	24	86	**95**	**96**	**96**	**100**	**100**	**100**
Wafer	**100**	**100**	**100**	**100**	89	89	**99**	**100**	**96**	**96**	**100**	**100**	**100**
*Our case-study (Section 5)*	*95*		*100*		*na*		*99*		*99*		*100*		*100*

**Table 3:** Robustness under swapping perturbations.

Dataset	mlp	Resnet	Mcdcnn	cnn	fcn	WEASEL
Car	na	✗	na	na	✗	na
DodgerLoopWeekend	✓	✗	✓	✓	✗	✓
FordA	na	✗	na	na	✗	✗
FreezerRegularTrain	na	✗	✗	✗	✗	✗
FreezerSmallTrain	na	✗	na	na	na	✗
ItalyPowerDemand	✗	✗	✗	✗	✗	✗
MoteStrain	na	✗	na	na	✗	✗
Plane	✗	✗	✗	✗	✗	✗
SonyAIBORobotSurface1	na	✗	na	na	✗	na
SonyAIBORobotSurface2	na	✗	na	na	✗	✗
StarLightCurves	na	✗	na	✓	✗	✗
Trace	na	✗	na	✗	✗	✗
Wafer	✓	✗	✓	✓	✗	✓

na: not considered in our study, ✓: robust (not impacted), ✗: not robust (impacted).

**Table 4:** Robustness under dropping perturbations.

Dataset	mlp	Resnet	Mcdcnn	cnn	fcn	WEASEL
Car	na	✗	na	na	✗	na
DodgerLoopWeekend	✓	✓	✓	✓	✓	✓
FordA	na	✓	na	na	✗	✗
FreezerRegularTrain	na	✓	✓	✓	✓	✓
FreezerSmallTrain	na	✓	na	na	na	✓
ItalyPowerDemand	✓	✓	✓	✓	✓	✓
MoteStrain	na	✓	na	na	✓	✓
Plane	✓	✓	✓	✓	✓	✓
SonyAIBORobotSurface1	na	✓	na	na	✗	na
SonyAIBORobotSurface2	na	✓	na	na	✓	✓
StarLightCurves	na	✓	na	✓	✓	✓
Trace	na	✓	na	✓	✓	✓
Wafer	✓	✓	✓	✓	✓	✓

na: not considered in our study, ✓: robust (not impacted), ✗: not robust (impacted).

**Table 5:** Dataset characteristics for decision trees.

Dataset	*len(TS)*	*No_cl_*	$P_{\mathrm{in}-\mathrm{cl}}$	$P_{\mathrm{bet}-\mathrm{cl}}$	Deriv
Car	577	4	0.862	0.826	0.99
DodgerLoopWeekend	288	2	0.72	0.585	0.569
FordA	500	2	0.002	−0.002	0.115
FreezerRegularTrain	301	2	0.799	0.715	0.629
FreezerSmallTrain	301	2	0.799	0.715	0.629
ItalyPowerDemand	24	2	0.829	0.734	0.236
MoteStrain	84	2	0.547	0.421	0.238
Plane	144	7	0.95	0.648	0.131
SonyAIBORobotSurface1	70	2	0.743	0.672	0.153
SonyAIBORobotSurface2	65	2	0.402	0.231	0.206
Trace	275	4	0.743	0.064	0.575
Wafer	152	2	0.123	0.044	0.386
*Our case-study (Section 5)*	*305*	*3*	*0.7180*	*0.5470*	*0.0459*

**len(TS)**: the time-series length, **$No_{cl}$**: the number of classes, **$P_{in-cl}$**: the Pearson correlation coefficients between all time-series of the same class, **$P_{bet-cl}$**: the Pearson correlation coefficients between the different classes, **Deriv**: the average derivative.

**Table 6:** Characteristics of decision trees for both perturbations.

Classifier	Swapping Perturb.		Dropping Perturb.	
	Leaves	Depth	Leaves	Depth
mlp	21	7	1	0
resnet	125	12	6	5
mcdcnn	42	10	1	0
cnn	68	10	1	0
fcn	76	12	10	5
weasel	64	10	2	1

**Table 7:** Accuracy of decision trees for the swapping effect.

Classifier	mlp	resnet	mcdcnn	cnn	fcn	weasel
Swapping perturb.	12%	30%	43%	37%	36%	89%
Dropping perturb.	100%	80%	100%	100%	100%	100%

## Data Availability

Not applicable.
